# SARS-CoV-2 Infection in Pregnant Women and Their Newborns

**DOI:** 10.5334/aogh.3072

**Published:** 2020-10-08

**Authors:** Melanie Etti, Musa Sekikubo, Victoria Nankabirwa, Halvor Sommerfelt, Bridget Freyne, Kondwani Kawaza, Gladys Gadama, Kondwani Jambo, Esperança Sevene, Marleen Temmerman, Laura A. Magee, Peter von Dadelszen, Asma Khalil, Kirsty Le Doare, WHO/HRP Alliance author group

**Affiliations:** 1 Makerere University Johns Hopkins University Research Collaboration, Kampala, UG; 2 Paediatric Infectious Diseases Research Group, Institute for Infection and Immunity, St George’s, University of London, UK; 3 Department of Obstetrics and Gynaecology, Makerere University, College of Health Sciences, Kampala, UG; 4 Department of Epidemiology and Biostatistics, Makerere University, College of Health Sciences, Kampala, UG; 5 Center for Intervention Science in Maternal and Child Health (CISMAC), University of Bergen, NO; 6 Malawi-Liverpool-Wellcome Trust Clinical Research Programme and Institute of Infection and Global Health, University of Liverpool, UK; 7 Department of Paediatrics and Child Health, University of Malawi, Blantyre, MW; 8 Department of Obstetrics and Gynaecology, Queen Elizabeth Central Hospital and College of Medicine, University of Malawi, MW; 9 Viral Immunology Research Group, Malawi-Liverpool-Wellcome Trust Clinical Research Programme, Blantyre, MW; 10 Manhiça Health Research Centre & Faculty of Medicine, Eduardo Mondlane University, Maputo, MZ; 11 Centre of Excellence in Women, Child and Adolescent Health, Aga Khan University, Nairobi, KE; 12 School of Life Course Sciences, Faculty of Life Sciences & Medicine, King’s College London, UK; 13 Fetal Medicine Unit, Department of Obstetrics and Gynaecology, St. George’s University Hospitals NHS Foundation Trust, London, UK; 14 WHO/HRP Alliance author group, CH

## Abstract

There remain a number of uncertainties globally about the risks posed to women who are infected with SARS-CoV-2 during pregnancy. Furthermore, our understanding of the spread of COVID-19 in Sub-Saharan Africa is limited, owing to low testing rates in many parts of the continent. PeriCOVID Africa, in conjunction with the WHO/HRP Alliance, plans to address these knowledge gaps by harnessing research infrastructures in place in five sub-Saharan African countries in order to screen more than 50,000 pregnant women and their infants for SARS-CoV-2, while monitoring pregnancy and neonatal outcomes. We anticipate that the results of this study will provide much needed information about the risks that SARS-CoV-2 poses to pregnant women and their babies, as well as establishing potential routes of mother-to-child transmission.

To the Editor,

There remain a number of uncertainties globally about the risks posed to women who are infected with SARS-CoV-2 during pregnancy, in particular, whether COVID-19 increases the risk of adverse outcomes for mother and baby, and the ways in which the virus or immunity to the virus may be passed from the pregnant woman or breastfeeding mother to her infant. Much of the strongest evidence published thus far has been in the form of systematic reviews and meta-analyses of observational data [1,2,3], and little has been published on this topic from Africa to date.

There have been a number of registries set up internationally to capture data from women who have been diagnosed with COVID-19 during pregnancy, many of which have been instrumental in providing crucial observational data about pregnancy outcomes in this cohort. These data are, however, limited in that they do not provide sufficient insight into the mechanisms underlying the observed outcomes, highlighting a need for the collection of clinical samples in this context.

Recently-published data have also suggested that symptomatic women may represent only the tip of the iceberg of SARS-CoV-2 infection in pregnancy [4,5], making it possible that registries contain data from only a small proportion of true SARS-CoV-2 positive cases, especially in countries where mass testing has not yet been adopted. It is therefore imperative that asymptomatic pregnant women are also screened for the virus in order to capture the full breadth of effects of SARS-CoV-2 infection in both pregnancy and infancy.

PeriCOVID Africa plans to address these knowledge gaps by harnessing the infrastructures already in place for a number of large studies in sub-Saharan Africa, including PREPARE, PRECISE, BAMBI II and the “Early versus late BCG vaccination in HIV-1 exposed infants in Uganda” randomised controlled trial. This platform will screen more than 59,000 pregnant women for SARS-CoV-2 infection and collect clinical samples and data from mothers and babies among a birth cohort of nearly 100,000 women and infants in the Gambia, Kenya, Malawi, Mozambique and Uganda, with additional data from collaborators in South Africa (Figure 1).

**Figure 1:**
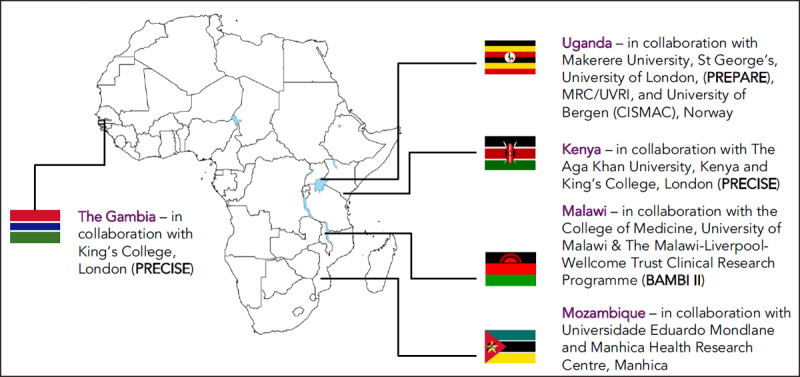
Research collaborations in sub-Saharan Africa recruiting participants for periCOVID Africa.

This body of work will be carried out in conjunction with the World Health Organisation (WHO) and the United Nations Development Program (UNDP)-United Nations Population Fund (UNFPA)-UNICEF-WHO-World Bank Special Program of Research, Development and Research Training in Human Reproduction, known as the HRP Alliance, who together hosted COVID-19 and pregnancy research working group meetings which brought together global expertise, enabling the development a generic cohort protocol addressing COVID-19 among pregnant women and their neonates.

Over a 12-month period, we will collect throat swabs (to test for SARS-CoV-2 RNA) and blood samples (for SARS-CoV-2 serology [IgM/IgG]) from pregnant women presenting for antenatal care at our designated study sites, based in primary health care facilities and government hospitals in each of the five countries. In alignment with the WHO/HRP Alliance’s generic cohort protocol, we will then follow these women until delivery where we will take further samples including cord blood and nasal swabs from the infants to assess for evidence of vertical transmission, and will also collect clinical data from all enrolled women to determine the relative risk of adverse outcomes in women who test positive for SARS-CoV-2 during pregnancy. All data collected will be integrated into existing registries and reported monthly to the WHO/HRP Alliance-designated platform so that important results can be widely disseminated in real-time.

We anticipate that the results of this study will help provide the first vital steps to understanding the pathophysiology of the risks that SARS-CoV-2 poses to pregnant women and their babies, as well as establishing potential routes of mother-to-child transmission, which will inform infection prevention and control measures recommended for pregnant and breastfeeding women.

## References

[1] Della Gatta AN, Rizzo R, Pilu G, Simonazzi G. Coronavirus disease 2019 during pregnancy: A systematic review of reported cases. Am J Obstet Gynecol. 2020; 223(1): 36–41. DOI: 10.1016/j.ajog.2020.04.013 32311350 PMC7165087

[2] Walker KF, O’Donoghue K, Grace N, et al. Maternal transmission of SARS-CoV-2 to the neonate, and possible routes for such transmission: A systematic review and critical analysis. BJOG An Int J Obstet Gynaecol. 2020; 1–13. DOI: 10.1111/1471-0528.16362 PMC7323034 32531146

[3] Khalil A, Kalafat E, Benlioglu C, et al. SARS-CoV-2 infection in pregnancy: A systematic review and meta-analysis of clinical features and pregnancy outcomes. EClinicalMedicine; 2020 DOI: 10.1016/j.eclinm.2020.100446 PMC7334039 32838230

[4] Khalil A, Hill R, Ladhani S, Pattisson K, O’Brien P. SARS-CoV-2 in pregnancy: Symptomatic pregnant women are only the tip of the iceberg. Am J Obstet Gynecol; 2020 DOI: 10.1016/j.ajog.2020.05.005 PMC7204681 32387327

[5] Sutton D, Fuchs K, D’Alton M, Goffman D. Universal screening for SARS-CoV-2 in women admitted for delivery. N Engl J Med. 2020; 382(22): 2163–64. DOI: 10.1056/NEJMc2009316 32283004 PMC7175422

